# An association study between *Heme oxygenase-1* genetic variants and Parkinson's disease

**DOI:** 10.3389/fncel.2014.00298

**Published:** 2014-09-24

**Authors:** Pedro Ayuso, Carmen Martínez, Pau Pastor, Oswaldo Lorenzo-Betancor, Antonio Luengo, Félix J. Jiménez-Jiménez, Hortensia Alonso-Navarro, José A. G. Agúndez, Elena García-Martín

**Affiliations:** ^1^Department of Biochemistry, Molecular Biology, and Genetic, University of ExtremaduraCáceres, Spain; ^2^Redes Temáticas de Investigación Cooperativa en Salud (RIRAAF/RETICS), Instituto de Salud Carlos IIIMadrid, Spain; ^3^Department of Pharmacology, University of ExtremaduraCáceres, Spain; ^4^Neurogenetics Laboratory, Division of Neurosciences, Center for Applied Medical Research (CIMA), University of NavarraPamplona, Spain; ^5^Department of Neurology, School of Medicine, Clínica Universidad de Navarra, University of NavarraPamplona, Spain; ^6^Centro de Investigación Biomédica en Red de Enfermedades Neurodegenerativas (CIBERNED), Instituto de Salud Carlos IIIMadrid, Spain; ^7^Section of Neurology, Hospital Universitario del SuresteMadrid, Spain

**Keywords:** Parkinson's disease, Heme oxygenase, polymorphisms, copy number variations, biomarkers, blood-brain barrier, iron homeostasis

## Abstract

The blood-brain barrier (BBB) supplies brain tissues with nutrients, filters harmful compounds from the brain back to the bloodstream, and plays a key role in iron homeostasis in the human brain. Disruptions of the BBB are associated with several neurodegenerative conditions including Parkinson's disease (PD). Oxidative stress, iron deposition and mitochondrial impaired function are considered as risk factors for degeneration of the central nervous system. Heme oxygenase (HMOX) degrades heme ring to biliverdin, free ferrous iron and carbon monoxide being the rate-limiting activity in heme catabolism. The isoform HMOX1 is highly inducible in response to reactive oxygen species, which induce an increase in BBB permeability and impair its pathophysiology. Consequently, an over- expression of this enzyme may contribute to the marked iron deposition found in PD. We analyzed the *HMOX1* SNPs rs2071746, rs2071747, and rs9282702, a microsatellite (GT)_*n*_ polymorphism and copy number variations in 691 patients suffering from PD and 766 healthy control individuals. Copy number variations in the *HMOX1* gene exist, but these do not seem to be associated with PD risk. In contrast two polymorphisms that modify the transcriptional activity of the gene, namely a VNTR (GT)_*n*_ and the SNP rs2071746, are strongly associated with PD risk, particularly with the classic PD phenotype and with early onset of the disease. This study indicates that *HMOX1* gene variants are associated to the risk of developing some forms of PD, thus adding new information that supports association of *HMOX* gene variations with PD risk.

## Introduction

The blood-brain barrier (BBB) supplies brain tissues with nutrients, filters harmful compounds from the brain back to the bloodstream, and plays a key role in iron homeostasis in human brain. Disruptions of the BBB are associated with several neurodegenerative diseases including Parkinson's disease (PD). Oxidative stress, iron deposition and mitochondrial impaired function are considered as risk factors for degeneration of the central nervous system (Schipper, 2004; Alonso-Navarro et al., 2008). In addition, it has been postulated that BBB inflammation may be related to PD risk by causing over-activation of microglia, increased production of cytokines and release of reactive oxygen species (Whitton, 2007), and the possibility that PD may be related to blood borne toxins could be the cause of these neurodegenerative disorders if the integrity of BBB is compromised (for a recent review, see Kim et al., 2012).

PD is one of the most common neurodegenerative movement disorders; current demographic trends indicate a lifetime risk approaching 4% (Schapira, 2013). The mean age of onset is 70 years, although 4% of patients develop early-onset, before the age of 50 (Schrag and Schott, 2006). This disease is characterized by rest tremors, rigidity, slowness of movement and postural imbalance. The primary pathologic abnormalities are the loss of the pigmented cells of the substancia nigra pars compacta, and the dopaminergic neurons of the striatum, which decreases in dopamine levels (Lu'o'ng and Nguyen, 2012). Other central and peripheral dopaminergic and non-dopaminergic systems are also involved. Regarding the pathogenesis of neurodegeneration in PD, patients show increased levels of oxidized lipids and a decrease in levels of glutathione (Zeevalk et al., 2008; Obeso et al., 2010; Schapira, 2012). In these patients, concentration of phospholipids and polyunsaturated free fatty acids, which are highly susceptible to oxidants, is decreased, whereas malondialdehyde, a marker of lipid oxidation, is increased (Dexter et al., 1989; Zhou et al., 2008). Several studies have suggested the occurrence of genetic factors related to PD risk, but so far no major genetic risk factors have been identified, and it is widely accepted that PD is likely to be related to genetic plus environmental interaction (Schrag and Schott, 2006; Obeso et al., 2010; Trinh and Farrer, 2013; Alonso-Navarro et al., 2014) and that further genetic association studies based on solid functional mechanisms related to the pathogenesis of the disease are required.

A relevant feature of the development of PD is abnormal iron deposition in the substantia nigra of PD patients, as has been demonstrated both in post-mortem studies (Sofic et al., 1988) and in *in vivo* studies (Hochstrasser et al., 2004; Berg and Hochstrasser, 2006). Some consequences of elevated nigral iron levels are well known. The intranigral administration of iron produces selective dopaminergic neuronal loss and elicits parkinsonian symptoms (Youdim, 2003). Alteration of iron homeostasis, dysfunction in molecules involved in sequestering excess iron, as well as ageing, are considered as possible causes of elevated iron deposition. BBB protects the brain against circulating iron, however the specific mechanisms involved in the regulation of iron release and transport that lead to iron accumulation in aging and neurological disorders such as PD, are not completely understood. The involvement of dysregulation of brain iron homeostasis in PD has been recently revised (Weinreb et al., 2013). It was suggested that alterations in brain vascularization in certain areas may cause high levels of iron (Faucheux et al., 1999).

Heme oxygenase (HMOX) enzyme activity degrades heme ring to biliverdin, free ferrous iron and carbon monoxide being the rate-limiting activity in heme catabolism (Ryter and Tyrrell, 2000). Two HMOX isoforms have been described in the mammalian central nervous system: HMOX1 is present in neurons and neuroglia (Benjamini and Hochberg, 1995), and it is inducible or repressible by pro-oxidant and other microenvironment stimuli (Kinobe et al., 2006; Loboda et al., 2008). HMOX2 is ubiquitously and constitutively expressed throughout the mammalian neuraxis, predominantly in substantia nigra, septum and hippocampus (Verma et al., 1993; Maines, 1997). A third isoform unique to rats, designated as HMOX3, exists (Scapagnini et al., 2002). The role of single nucleotide polymorphisms (SNPs) in the *HMOX2* gene in patients with PD has been reported, the *HMOX2* SNP rs2270363 being associated with PD risk (Ayuso et al., 2011). In contrast, little is known about the role of genetic variations in the *HMOX1* gene in PD, and controversial findings justify the analysis of this gene in a large sample population (Funke et al., 2009; Infante et al., 2010). *HMOX1* is a plausible candidate gene for PD risk because immunoreactive HMOX1 has been identified in dopaminergic neurons of PD patients (Castellani et al., 1996; Schipper, 1998). In addition, neurotoxins, cytokines and nitric oxide are considered as inducers of astroglial *HMOX1* expression (Rieder et al., 2004; Schipper, 2004). Moreover, under stress conditions in astroglia, HMOX1 promotes mitochondrial sequestration of non-transferrin iron and may contribute to the pathological iron deposition described in PD (Schipper et al., 2009). In this study we analyze common variations and copy number variations in the *HMOX1* gene, selected according to their allelic frequencies and putative functional effect on enzyme activity, in a large sample of patients with PD and healthy controls.

## Methods

Subjects were recruited from several centers in Spain. The study group was composed of 691 PD patients and 766 healthy controls, which were included in the study after obtaining informed consent. From these, 460 patients with PD and 459 control individuals were recruited from the Clínica Universidad de Navarra (Pamplona, Spain). In addition, 231 patients with PD were recruited from the Hospital de La Princesa (Madrid, Spain) and 307 controls were included from the latter hospital and from the Infanta Cristina University Hospital (Badajoz, Spain). Most participants had been included in previous studies by our group (Agundez et al., 2008a; Garcia-Martin et al., 2008; Ayuso et al., 2011; Lorenzo-Betancor et al., 2011; Jimenez-Jimenez et al., 2014). All the participants were Caucasian Spanish individuals as stated in the self-report. All consecutive patients diagnosed by consultant neurologists according to the criteria recommended by Hughes et al. (1992) were requested to participate and all of them agreed to do so. Selection criteria for patients with PD included bradykinesia and at least one of the following symptoms: rigidity, resting tremor, postural instability, a positive response to dopaminergic therapy, and the absence of atypical features or other causes of parkinsonism (Hughes et al., 1992). From the total of patients, only 36 of the 691 patients with PD had a positive family history of the disease and monogenic forms of PD were excluded. Patients were divided in three groups according to different phenotypes: tremor-dominant patients with PD (TD-PD), classical phenotype patients with PD (C-PD), or akinetic-rigid patients with PD (AR-PD) when such information was available (Lorenzo-Betancor et al., 2011). As the classification of motor PD phenotype subgroups can be arbitrary, we considered those individuals with rest tremor as dominant and an initial feature of their disease to have a TD-PD. AR-PD phenotype was considered for those individuals with predominant bradykinesia or signs of rigidity, but mild or no tremor at rest. The patients with comparable severity of tremor, rigidity, and bradykinesia were considered patients with C-PD. A medical examination was performed to identify individuals in good health. The study was approved by the ethics committees of the institutions involved in the study. For all participants, genomic DNA was obtained from peripheral leukocytes and purified according to standard procedures. Table 1 summarizes the demographic data of the subgroups analyzed in the study.

**Table 1:** **Demographic data of the sample analyzed in this study**.

**Group**	**Controls (n = 766)**	**PD**				
		**Entire PD (n = 691)**	**TD-PD (n = 30)**	**C-PD (n = 328)**	**AR-PD (n = 94)**	**Unclassifiable PD^a^ (n = 239)**
Age, y, mean (*SD*)	64.67 (14.28)	67.09 (10.62)	69,51 (8.09)	67.06 (10.68)	66.00 (10.79)	67.32 (11.76)
Age range, y	17–102	22–95	49–87	22–95	39–87	41–88
AAO, y, mean (*SD*)	NA	59.57 (12.61)	62.82 (8.13)	57.55 (11.58)	56.81 (11.59)	63.51 (13.65)
AAO range, y	NA	17–90	46–76	17–84	34–84	18–90
Female %	45.7%	42.6%	43.3%	36.8%	42.5%	50.2%

*PD, Parkinson's disease; y, years; AAO, age at onset; SD, standard deviation; NA, not available; TD-PD, tremor-dominant Parkinson's disease; C-PD, classical PD phenotype; AR-PD, Akinetic-rigid PD*.

a *PD subjects with no information on motor features available*.

Four polymorphisms in the *HMOX1* gene, located in chromosome 22q12, were selected for genotyping on the basis of putative functional effects as well as expected allele frequency in Caucasian individuals as reported in public databases (http://www.ncbi.nlm.nih.gov/projects/SNP/snp_ref.cgi?showRare=on&chooseRs=all&go=Go&locusId=3162). These polymorphisms included a variable number tandem repeat (VNTR), consisting of alternating the purine-pyrimidine sequence (GT)_*n*_ which has the potential to acquire the Z-DNA conformation, a structure which is thermodynamically unfavorable compared with the B-DNA structure. This formation has been described as a factor that negatively affects transcriptional activity (Naylor and Clark, 1990; Delic et al., 1991) and that causes differences in transcriptional activity of the promoter of *HMOX1* with different numbers of repeats. For the association study of the *HMOX1* GT_(*n*)_, the alleles were divided into two classes: class S including short repeat sequences (*n* < 25) and class L including long repeat sequences (*n* ≥ 25) (Yamada et al., 2000; Tiroch et al., 2007). The presence of this VNTR polymorphism was analyzed after PCR amplification (forward primer 5′-AGAGCCTGCAGCTTCTCAGA-3′ and reverse primer 5′-CTCTGGCTTCCTAGCAGGG-3′) and quantification of number of repetitions by using GeneGel HyRes Denaturing gels, and DNA staining kit Silver staining kit (GE Healthcare, Madrid, Spain).

Three single nucleotide polymorphisms were studied by means of TaqMan probes (Applied Biosciences Hispania, Alcobendas, Madrid, Spain): The SNP rs2071746 (A/T) is an upstream variant located at 22:35380679, rs2071747 is a missense mutation G/C (Asp/His) located at 22: 35381192, which corresponds to exon 1 of the *HMOX1* gene, and rs9282702 consists of a missense mutation C/T (Pro/Leu) located at 22:35386857, which corresponds to exon 3 of the *HMOX1* gene. Besides these SNPs, no other non-synonymous *HMOX1* SNPs have been reported to occur in Caucasian individuals at frequencies over 1%. Custom primers were designed to analyze the SNPs rs9282702 and rs2071747, whereas commercial primers were used for the detection of the SNPs rs2071746 (C__15869717_10, Applied Biosciences Hispania, Alcobendas, Madrid, Spain). The detection was carried out by qPCR in an Eppendorf Realplex thermocycler by using fluorescent probes. The amplification conditions were as follows: After a denaturation time of 10 min at 96°C, 45 cycles of 92°C 15 s 60°C 90 s were carried out and fluorescence was measured at the end of each cycle and at endpoint. All samples were determined in triplicate and genotypes were assigned both by gene identification software (RealPlex 2.0, Eppendorf) and by analysis of the reference cycle number for each fluorescence curve, calculated by the CalQPlex algorithm (Eppendorf). For technical validation purposes, the amplified fragments for 20 individuals carrying every genotype (AA, AT and TT for rs2071746 and GG, GC and all carriers of the CC genotype for rs2071747 were sequenced, and in all cases the genotypes fully corresponded with those detected with fluorescent probes.

Copy number variations (CNVs) of the *HMOX1* gene were analyzed using the TaqMan copy number assay Hs00774483_cn (Applied Biosciences Hispania), which was designed to hybridize within the open reading frame in *HMOX1* gene exon 3 (chromosome location 22:35782935). Amplification was carried out in an Applied Biosystems 7500 real-time thermocycler as described by the manufacturer, using as a copy number reference assay RNAse P. All reactions were carried out in quadruplicate. Results were analyzed by means of the CopyCaller Software (Applied Biosciences Hispania). According to standard procedures in CNV analyses, we designed as heterozygous (null/present) those samples with a single copy of the *HMOX1* gene.

## Statistical analysis

The Hardy–Weinberg equilibrium was analyzed with the DeFinetti program (http://ihg.gsf.de/cgi-bin/hw/hwa1.pl). Allelic and genotype analyses were performed with the PLINK software (Purcell et al., 2007). Haplotype reconstruction was performed using the program PHASE v2.1.1 (Stephens et al., 2001). This reconstruction was carried out to detect putative association of at risk haplotypes. We used the default model for recombination rate variation with 1000 iterations, 500 burn-in iterations, and a thinning interval of 1. Diplotypes were obtained from the combination of haplotypes in the best run (the one that showed the maximum consistency of results across all runs); further details are provided elsewhere (Agundez et al., 2008b). Statistical analyses were performed using the SPSS 15.0 for Windows (SPSS Inc., Chicago, Illinois, USA). Intergroup comparison values were calculated by using the χ^2^ or Fisher tests when appropriate. The 95% confidence intervals were also calculated. The threshold for statistical significance was *p* < 0.05. Correction for multiple testing was done according the false discovery rate (FDR) procedure as described elsewhere (Benjamini and Hochberg, 1995). The statistical power was determined from variant allele frequencies observed in control individuals with a genetic model analyzing the frequency for carriers of the disease gene with a relative risk (RR) value = 1.5 (*p* = 0.05). The statistical power for two-tailed associations for the presence of any of the two SNPs identified in this study was 99.9% for overall patients with PD, 98.3% for patients with C-PD and 95.4% for patients with an unknown PD phenotype. Testing for heterogeneous association (homogeneity test) was analyzed by using the Breslow–Day test.

## Results

At the first stage, because of DNA shortage, we analyzed the occurrence of the four *HMOX1* gene polymorphisms, including the VNTR and the three SNPs described under methods, in 100 randomly selected samples from PD patients and 150 samples from healthy individuals. The SNP rs9282702 was monomorphic. The rest of gene variations analyzed were found to be polymorphic in the population study, and therefore we extended the analyses to all patients and control individuals participating in the study. CNV analyses in the whole study group revealed the occurrence of three patients with PD and six control individuals with a single copy of the *HMOX1* gene. All these individuals were carriers of one null allele in heterozygosity. Individuals with zero or more than two gene copies were not identified in the study group. To our knowledge this is the first description of the occurrence of CNVs in the *HMOX1* gene, but the occurrence of CNVs does not seem to have a major association with PD risk.

The combined genotypes obtained after SNP and CNV analyses are summarized in Table 2. No deviation from Hardy–Weinberg equilibrium was found for the SNPs or CNVs analyzed either in the subgroups of patients suffering from PD or in the control group. The risk of PD associated with the SNPs and CNVs was estimated considering the major allele as the risk allele, by comparison of allelic, heterozygous, and homozygous dominant and recessive models. The major alleles for the SNPs rs2071746 and rs2071747 were A and G, respectively, in agreement with the allele frequencies shown in the 1000 genomes catalog for European individuals (http://browser.1000genomes.org/Homo_sapiens/Info/Index). The best fit was observed with the dominant and the allelic models. We identified a statistically significant difference of the rs2071746 genotypes between patients with C-PD and healthy individuals. This difference was observed both in the dominant model and in the allelic model. After correction for the three PD presentations (TD-PD, C-PD, and AR-PD), these associations remained statistically significant in the C-PD group. In addition, a weak statistically significant difference in the frequency of *HMOX1* rs2071746 genotypes between patients with TD-PD and healthy individuals was observed. This difference followed a dominant model, but the difference was not statistically significant in the allelic model (Table 2). When the 20 patients with C-PD and positive family history of PD were excluded, the statistical significance of the OR values were similar (not shown). No association with the SNP rs2071747 was identified in any of the PD phenotypes. The SNPs, rs2071746, and rs2071747 did not show linkage disequilibrium (*D*′ = 0.652, *r* = 0.147), and no particular risk haplotypes were identified in any of the study groups.

**Table 2:** ***HMOX1* genotype and allele frequencies in patients with Parkinson's disease according to phenotypes**.

**SNP**	**Patients**	**Patients subgroups**	**No**.	**Genotype frequencies (%)**					**P (dominant model)**	**OR (95% CI)**	**Allele frequencies (%)**		**P (allelic model)**	**OR (95%CI)**
**rs2071746**				**A/A**	**A/T**	**T/T**	**Null/A**	**Null/T**			**A**	**T**		
	PD		691	31.2	47.6	20.6	0.4	0	0.019	0.76 (0.61–0.96)	55.1	44.9	0.054	0.87 (0.75–1.00)
		TD-PD	30	44.8	27.6	27.6	0	0	0.010; *Pc* = 0.040	0.39 (0.18–0.81)	60.0	40.0	0.193; *Pc* = 0.308	0.71 (0.42–1.20)
		C-PD	328	35.3	47.9	16.5	0.3	0	0.0009; *Pc* = 0.007	0.62 (0.47–0.83)	59.3	40.7	0.0006; *Pc* = 0.005	0.72 (0.60–0.87)
		AR-PD	94	35.6	49.4	13.6	1.4	0	0.045; *Pc* = 0.090	0.63 (0.40–0.99)	59.6	40.4	0.017; *Pc* = 0.045	0.69 (0.51–0.94)
		Unknown phenotype	239	21.3	49.2	29.5	0	0	0.110; *Pc* = 0.176	1.26 (0.89–1.79)	46.0	54.0	0.039; *Pc* = 0.078	1.24 (1.01–1.53)
	Controls		766	25.4	51.2	22.6	0.3	0.5	Ref	Ref	51.0	49.0	Ref	Ref
**rs2071747**				**G/G**	**G/C**	**C/C**	**Null/G**	**Null/C**			**G**	**C**		
	PD		691	92.5	6.9	0.2	0.4	0	0.172	0.77 (0.53–1.12)	96.0	4.0	0.163	0.78 (0.54–1.11)
		TD-PD	30	90.0	10.0	0.0	0	0	0.931; *Pc* = 0.931	1.06 (0.31–3.56)	95.0	5.0	1.000; *Pc* = 1.000	1.02 (0.31–3.34)
		C-PD	328	91.5	8.2	0.0	0.3	0	0.603; *Pc* = 0.804	0.89 (0.56–1.40)	95.6	4.4	0.525; *Pc* = 0.653	0.87 (0.56–1.35)
		AR-PD	94	89.3	7.9	1.3	1.5	0	0.731; *Pc* = 0.835	1.13 (0.56–2.27)	93.1	6.9	0.571; *Pc* = 0.653	1.21 0.63–2.32)
		Unknown phenotype	239	95.5	4.5	0.0	0	0	0.016; *Pc* = 0.043	0.46 (0.24–0.88)	97.7	2.3	0.014; *Pc* = 0.045	0.46 (0.24–0.87)
	Controls		766	90.4	8.5	0.3	0.7	0.0	Ref	Ref	94.7	5.3	Ref	Ref

*TD-PD, tremor-dominant Parkinson's disease; C-PD, classical PD phenotype; AR-PD, Akinetic-rigid PD; Null, Null allele according to CNV analysis; Ref, reference group; Pc, P-value after correction for multiple comparisons (FDR, eight comparisons; two SNPs and four clinical groups)*.

As has been described previously, patients with the phenotypes TP-PD and C-PD may have an earlier age at the onset than the AR-PD group (Rajput et al., 2009), so we analyzed whether *HMOX1* rs2071746 genotype frequencies were related to age of onset in the C-PD subgroup. Patients with C-PD phenotype were subdivided according to the median age at onset in this subgroup (62 years). Table 3 summarizes these results. We observed statistically significant differences among patients with early onset of C-PD both, in the dominant model and in the allelic model, whereas such significant differences were not observed among patients with late-onset C-PD.

**Table 3:** ***HMOX1* rs2071746 genotypes in C-PD subjects stratified according to age of onset**.

**SNP**	**Patients**	**Patients subgroups**	**No**.	**Genotype frequencies (%)**					**P (dominant model)**	**OR (95% CI)**	**Allele frequencies (%)**		**P (allelic model)**	**OR (95%CI)**
**rs2071746**				**A/A**	**A/T**	**T/T**	**Null/A**	**Null/T**			**A**	**T**		
	C-PD	Onset < 62 years	187	37.4	46.5	15.5	0.5	0.0	0.0011; *Pc* = 0.0022	0.57 (0.41–0.80)	60.9	39.0	0.0009; *Pc* = 0.0018	0.68 (0.54–0.85)
	C-PD	Onset ≥ 62 years	138	33.3	47.8	18.8	0.0	0.0	0.056; *Pc* =0.056	0.68 (0.46–1.01)	57.2	42.8	0.075; *Pc* = 0.075	0.79 (0.61–1.03)
	Controls		766	25.4	51.2	22.6	0.3	0.5	Ref	Ref	51.0	49.0	Ref	Ref

*Null, Null allele according to CNV analysis; Ref, reference group; Pc, P-value after correction for multiple comparisons (FDR, two comparisons; one SNP and two age groups)*.

Regarding the HMOX-1 GT_(*n*)_ polymorphism, we studied a subset of the total of patients and healthy subjects included in this study because of DNA shortage. Thus, 365 PD patients and 371 healthy subjects were genotyped. The number of repeats ranged from 11 to 33, with the highest frequency for GT_(20)_ repeats in healthy subjects and GT_(22)_ repeats in the case of PD patients. Mann–Whitney comparison for means in each group revealed statistically significant differences (*p* < 0.001). The association study of this polymorphism with PD patients is shown Table 4.

**Table 4:** **Genotype and allele frequencies for (GT)_*n*_ repeat polymorphisms in PD patients and healthy subjects**.

**Patients**	**Genotype frequencies (%)**			**P (dominant model)**	**OR (95% CI)**	**Allele frequencies (%)**		**P (allelic model)**	**OR (95%CI)**
	**S/S**	**S/L**	**L/L**			**S**	**L**		
PD (*N* = 365)	86.3	12.3	1.4	0.0001	0.34 (0.20–0.59)	0.92	0.08	0.0001	0.37 (0.23–0.62)
Controls (*N* = 371)	94.9	4.3	0.8			0.97	0.03	Ref	

*S, short repeat sequences (n < 25 (GT)n); L long repeats sequences (n ≥ 25 (GT)n)*.

## Discussion

BBB permeability is increased in the presence of reactive oxygen species, and it is widely accepted that the *HMOX1* mRNA upregulation in CNS is a marker of oxidative stress. The enhanced expression of *HMOX1* may contribute to the pathological deposition of iron which has been reported in normal aging CNS, and to a much greater extent in neurodegenerative disorders such as PD (Droge, 2002). Impaired HMOX1 activity caused by genetic variants could modify the intraneuronal production of biliverdin/bilirubin metabolites which exert an antioxidant role (Dore et al., 1999) contributing to the etiology of PD and other central nervous system disorders. Our group reported that functional variations in *HMOX2* gene are associated with PD risk (Ayuso et al., 2011). In this context and bearing in mind that HMOX1 has been postulated as a contributor to the pathological iron deposition described in PD (Schipper et al., 2009), we aimed to analyzed the possible role of *HMOX1* genetic variants in a large series of PD patients.

We identified a clinical association of the *HMOX1* rs2071746 polymorphism (A/T) with C-PD. The rs2071746 A allele was more common among PD patients with C-PD phenotype than in control individuals. Also, we observed that this association was related with an early onset of the disease. In addition, the analysis of the length of the highly polymorphic (GT)_*n*_ dinucleotide repeats indicates an association of longer (GT)_*n*_ repeats with susceptibility to PD. Both gene variations, rs2071746 and (GT)_*n*_ dinucleotide repeats sequence, have been related to variations in the transcriptional activity of the *HMOX1* gene (Yamada et al., 2000; Tanaka et al., 2011; Kramer et al., 2013). According published evidence, the number of (GT)_*n*_ repeats inversely correlates with *HMOX1* promoter activity and inducibility of the gene (Yamada et al., 2000), although controversy exist (Tanaka et al., 2011). It has been shown that the rs2071746 A allele is associated with high promoter activity (Ono et al., 2004). Our findings support an increased frequency of carriers of long (≥25 (GT)_*n*_) among PD patients and an increased frequency of carriers of the rs2071746 A allele among PD patients. Although these findings apparently point to opposite directions in terms of gene activity, the functional effect of these polymorphisms is not fully understood. Moreover, an extended model of the *HMOX1* gene along with the importance of alternative splicing in the area where these two polymorphisms are located, and its association with translational regulation has been disclosed recently (Kramer et al., 2013).

Our results agree with those by Kimpara et al. (1997) although other studies that failed to confirm these findings have been published (Funke et al., 2009). Putative discrepancy in the genetic associations may be explained because of the different ethnic origin of the participants included in each of these studies. Infante et al. (2010) reported association of the rs2071746 T allele in homozygosity as a risk factor for PD, but only when combined with another SNPs at the GSK3beta gene, and in a relatively small study group. In the study by Infante et al. however the rs2071746 T allele frequency is almost identical in patients and controls (39 and 37%). In fact, when the comparison of the rs2071746 genotypes or allele frequencies between patients and controls is made, no statistically significant differences are observed (Infante et al., 2010). In our study the association is statistically significant when analyzing genotypes or allele frequencies. Moreover, the large sample size analyzed in the present study and the fact that positive associations remained statistically significant after correction for multiple testing support the observed genetic associations. The effect of the rs2071746 SNP on different PD subgroups seems presenting consistent patterns, though some did not pass significance threshold. In fact, taking into consideration the dominant model (Table 2) the findings obtained in TD-PD and C-PD patients show different directions. Nevertheless it should be considered that the subgroup of TD-PD is composed of 30 patients only. These apparent discrepancies, and the lack of statistical significance in some subgroups, may be related to the small sample size of some of these subgroups. In addition, we analyzed for the first time the occurrence of CNVs in *HMOX1*, both in PD patients and in healthy individuals. We found CNVs more frequently in AR-PD patients. Although this study does not support a major role of *HMOX1* CNVs in PD risk, the demonstration of the occurrence of CNVs in the *HMOX1* gene supports further research in the functional role and clinical associations of *HMOX1* CNVs. It is of note, however, that the association cannot be observed in the subgroup of PD patients with unknown phenotype. Our findings suggest that the association of *HMOX1* genotypes and PD risk is specific of the C-PD phenotype. This reinforces the need of detailed phenotyping and categorization of patients in genetic association studies, particularly when assessing low-penetrance factors. In summary, in this study we identified two functional *HMOX1* gene variations as potential genetic biomarkers of PD. It is conceivable that these genetic biomarkers would be associated to other disorders related to oxidative stress in the CNS and in the BBB. This study adds to current knowledge of genetic biomarkers in PD (Alonso-Navarro et al., 2014) and other CNS disorders (Agundez et al., 2013) and, once these biomarkers are validated, these could be used to identify at risk individuals and to provide proof of concept regarding the mechanisms involved in the development of these diseases. Further research would be needed to analyze *HMOX1* genetic variations in an independent population to verify these associations and to analyze putative interethnic variability in the observed genetic association.

### Conflict of interest statement

The authors declare that the research was conducted in the absence of any commercial or financial relationships that could be construed as a potential conflict of interest.
